# On “Recommendations for Hospital-Based Physical Therapists Managing Patients with COVID-19.” Felten-Barentsz K, Van Oorsouw R, Klooster E, et al. *Phys Ther.* 2020;100:1444–1457. https://doi.org/10.1093/ptj/pzaa114

**DOI:** 10.1093/ptj/pzad037

**Published:** 2023-04-11

**Authors:** Karin M Felten-Barentsz, Juultje Sommers, Roel van Oorsouw, Emily Klooster, Niek Koenders, Linda van Heusden-Scholtalbers, Harm L Ormel, Maarten S Werkman, Erik H J Hulzebos, Philip J van der Wees, Marike van der Schaaf, Thomas J Hoogeboom

**Affiliations:** Radboud university medical center, Department of Rehabilitation, Nijmegen, the Netherlands; Amsterdam UMC, University of Amsterdam, Department of Rehabilitation Medicine, Amsterdam Movement Sciences, Meibergdreef 9, Amsterdam, the Netherlands; Radboud university medical center, Department of Rehabilitation, Nijmegen, the Netherlands; Deventer Hospital, Department of Rehabilitation, Deventer, the Netherlands; Radboud university medical center, IQ healthcare, Nijmegen, the Netherlands; Radboud university medical center, Department of Rehabilitation, Nijmegen, the Netherlands; Radboud university medical center, Department of Rehabilitation, Nijmegen, the Netherlands; Royal Dutch Society for Physical Therapy (KNGF), Quality Policy, Amersfoort, the Netherlands; Royal Dutch Society for Physical Therapy (KNGF), Quality Policy, Amersfoort, the Netherlands; Leiden University Medical Centre, Department of Physical Therapy, Leiden, the Netherlands; University Medical Centre Utrecht, Child Development and Exercise Centre, Utrecht, the Netherlands; Radboud university medical center, Department of Rehabilitation, Nijmegen, the Netherlands; Radboud university medical center, IQ healthcare, Nijmegen, the Netherlands; Amsterdam UMC, University of Amsterdam, Department of Rehabilitation Medicine, Amsterdam Movement Sciences, Meibergdreef 9, Amsterdam, the Netherlands; Amsterdam University of Applied Sciences, Centre of Expertise Urban Vitality, Faculty of Health, Amsterdam, the Netherlands; Radboud university medical center, Department of Rehabilitation, Nijmegen, the Netherlands; Radboud university medical center, IQ healthcare, Nijmegen, the Netherlands

With the surge of COVID-19 in China and seasonal fluctuations across the globe, it seems appropriate to share with *PTJ’s* readership the latest update of the 2020 Dutch recommendations published in *PTJ*^1^ regarding the management of patients hospitalized with COVID-19. This update was necessary due to an improved understanding of the disease, an increase in clinical expertise, and a growing body of scientific literature. To update the initial recommendations, we followed a pragmatic approach. First, we installed a working group comprising experts on content and guideline methodology. Subsequently, we consulted a group of 71 Dutch hospital-based physical therapists working with patients with COVID-19 to determine which recommendations should be updated and which topics should be added. For the highest 10 rated recommendations and topics, our working group conducted a pragmatic literature search. Finally, new and updated recommendations were formulated by merging the literature findings and the practical experiences. Below we address some of the salient updates.

## Safety Recommendations

Regarding the safety recommendations, we deem it no longer necessary to minimize contact with patients with COVID-19 because personal protective equipment (PPE) is sufficiently available in the Netherlands. If hospital-based physical therapists use the recommended PPE, as indicated by hospital policy, it is safe to work “hands on” with patients with COVID-19.

## Respiratory Support

We still recommend starting with inspiratory muscle training (IMT) in patients with evident clinical signs of inspiratory muscle weakness, perceived dyspnea, or both. Physical therapists should be aware that patients are suspected to be at risk for respiratory muscle weakness in the case of (prolonged) mechanical ventilation. This recommendation is supported by one study that evaluated the effects of IMT (pulmonary functions, dyspnea, functional performance, and quality of life) after weaning from mechanical ventilation in patients who recovered from COVID-19.^2^ No studies were found that evaluated the effects of expiratory muscle training, so this recommendation was removed due to a lack of evidence and clinical rationale. Finally, we recommend that physical therapists should consider the FITT-principles (Frequency, Intensity, Time, and Type) when prescribing breathing exercises to patients with COVID-19, to ensure the exercise is explicit and measurable.

## Active Mobilization While Patient Is Unconscious

We recommend that physical therapists should monitor and support passive range of motion when patients are sedated in the intensive care unit (ICU). When patients are lying in prone position, plexopathy is a high-risk complication caused by tension and compression of the brachial plexus. Contralateral head rotation and shoulder abduction beyond 90 degrees should be prevented when patients are lying in a swimmer’s position.^3^

We recommend that physical therapists need to be alert for the development of heterogenic ossifications. During the first wave of the pandemic, it was noted that patients with prolonged ICU stay due to COVID-19 developed heterogenic ossifications in their elbows, shoulders, knees and hips.^4^ A stiff, painful, and warm joint should alert physical therapists to consider the diagnosis of (being at risk of) heterogenic ossification. A radiograph or computed tomography scan might inform clinicians about relevant calcifications of the muscles. For the treatment of heterogenic ossifications, a rehabilitation physician should be consulted. If a heterogenic ossification is diagnosed, it is recommended that the patient exercise within a comfortable range of motion.

**Figure:**
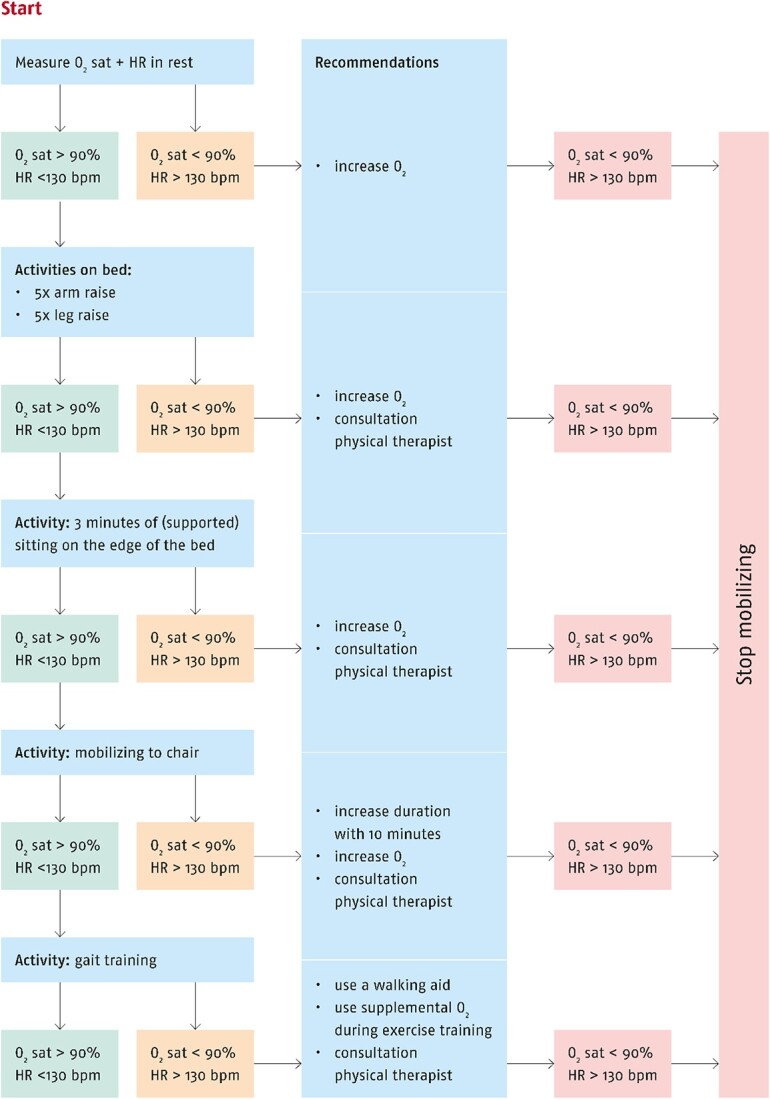
Flow chart for mobilization in patients with COVID-19. bpm = beats per minute; HR = heart rate; O_2_ = oxygen; O_2_ sat = oxygen saturation.

In the previous report of our recommendations, we were hesitant to recommend neuromuscular electrical stimulation (NMES) in sedated patients with COVID-19 in the ICU because of the lack of evidence of effectiveness, the hygienic challenges, the absence of the equipment in most Dutch hospitals, and our concerns about the feasibility during the hectic care of patients who are severely or critically ill. Recently, more scientific literature has become available supporting NMES as a potential treatment modality for patients with COVID-19 who are sedated and lying in a supine position.^5^ If electrical muscle contraction is possible, NMES might be helpful to reduce muscle atrophy in these patients.

## Active Mobilization While Patient Is Conscious and Able to Cooperate

We recommend that physical therapists should have an active role in mobilizing and activating patients on both the hospital ward and in cooperative patients in the ICU. Physical therapists should be aware that patients with COVID-19 are at risk to develop ICU-acquired weakness, even when they have no prior physical comorbidities. Experts in the field reported that patients with COVID-19 had disproportional rises in heart rate and strong deoxygenation during low-intensity active mobilization sessions. Patients experienced this low-intensity physical activity as extremely exhausting. As a result, physical therapists need to be aware of an increased risk of overload and exhaustion in this population. We still recommend that physical therapists should strictly follow safety criteria during their treatment.^1^ While performing active mobilization, we still recommend monitoring peripheral oxygen saturation, heart rate, and Borg Rating of Perceived Exertion score for perceived exhaustion in rest and during and after active mobilization.^1^ Ideally, active mobilization should be offered progressively based on the exercise response, with a maximum of 4 on the Borg scale.^1^ Finally, we recommend that physical therapists should advise nurses about mobilization principles in patients with COVID-19 (eg, using an expert-based flow chart (Figure).

In summary, our goal was to develop up-to-date recommendations based on new clinical insights, scientific literature, and best practices in the Netherlands that were feasible and acceptable in daily practice, facilitating their adoption and implementation. Although our recommendations overlap with other comprehensive, international acute care physical therapy guidelines for COVID-19,^6^ we believe that this letter offers practical tips and reminders at a time when COVID-19 is having a seasonal surge. We invite readers who are interested in receiving more detailed information about how we have applied and adapted our recommendations to contact the corresponding author.
